# Femtosecond Pulsed Fiber Laser Based on Graphdiyne-Modified Tapered Fiber

**DOI:** 10.3390/nano12122050

**Published:** 2022-06-15

**Authors:** Qing Wu, Si Chen, Wenli Bao, Haibin Wu

**Affiliations:** 1 Heilongjiang Province Key Laboratory of Laser Spectroscopy Technology and Application, Harbin University of Science and Technology, Harbin 150080, China; wuqing@hrbust.edu.cn; 2 Faculty of Basic Medicine, Hainan Vocational University of Science and Technology, Haikou 571126, China; chensics9@163.com; 3 School of Physics and Electronic Information, Gannan Normal University, Ganzhou 341000, China

**Keywords:** graphdiyne, tapered fiber, saturable absorber, femtosecond pulse

## Abstract

We report the application of saturable absorbers prepared from graphdiyne-modified tapered fibers to an erbium-doped fiber laser to achieve a femtosecond pulse output. Graphdiyne quantum dots are successfully prepared by the Glaser–Hay method. The graphdiyne-based all-fiber saturable absorber device exhibited strongly saturable absorption characteristics with a modulation depth of 18.06% and a saturation intensity of 103.5 W. The net dispersion of the erbium-doped fiber laser cavity is ~0.016 ps^2^, and a femtosecond pulse output with a bandwidth of 26.3 nm, a pulse width of 135.8 fs, and a single pulse capability of 54 pJ is obtained. This work lays the foundation for the application of the nonlinear optical material, graphdiyne, in ultrafast photonics.

## 1. Introduction

The all-fiber mode-locked laser cavity achieves an ultra-short pulse output, which has the advantages of high stability, high compatibility, high integration and high interference resistance [1,2]. Fiber lasers are more advantageous and attractive than solid-state lasers in terms of space occupied, economic efficiency, energy consumption, integration, and output performance. Ultrafast fiber laser technology has matured and facilitated many applications, such as fiber sensing technology, high-power lasers, and wavelength division multiplexing fiber communication systems. All-fiber light sources offer advantages such as environmental robustness, compactness, system compatibility and adaptability. The most common way to achieve mode-locking in fiber lasers is passive mode-locking, where saturable absorber (SA)-based fiber lasers are simpler to implement [3,4].

The earliest two-dimensional (2D) material used in SA is graphene, which was applied in an erbium-doped fiber laser in 2009 [5]. The graphene-based SA device with a “sandwich” structure was used in an erbium-doped fiber laser to achieve a mode-locked pulse output, so the graphene-based SA is known as one of the earliest 2D material-based photonic devices. Graphene has many advantages as SA devices, such as low saturation intensity, tunable modulation depth, and broadband tunability [5,6]. This has also opened the door for the exploration of 2D materials in saturable absorbers [7,8,9,10]. Many new 2D nanomaterials are becoming more and more abundant in optoelectronic research [11,12,13,14,15]. As a member of the carbon family, graphdiyne (GDY) is also of interest to researchers for its potential in the 1.5 μm band [16,17]. Graphene is formed by sp^2^ hybridization, while GDY is formed by sp and sp^2^ hybridization and is the most easily synthesized and stable isomer in the carbon family. The unique structure of GDY gives them great potential in the field of optics.

GDY, a new member of the family of two-dimensional materials, has attracted a great deal of attention from divergent research fields because of its outstanding merits, including carbon networks with delocalized π systems, due to the maintainability of their properties under clever variations of their electronic, optical, and geometrical properties. Using density functional theory calculations, a new series of alkali metal-adsorbed GDY structures (denoted as AM3@GDY (AM = Li, Na, K)) have an intramolecular electron donor-acceptor framework, which is sufficient for them to exhibit nonlinear optical behavior. Molecular structures containing a large number of p-conjugated networks can be designed as fundamental building blocks for exploring novel promising nonlinear materials, especially for two-dimensional structures, where the p-conjugation length and electron donor-acceptor processes of these species can largely enhance nonlinear optical properties. However, the optical nonlinearity and related applications of GDY have been little studied so far. Unlike graphene with a zero band gap, GDY is characterized by a natural band gap. Theoretically, the band gap of GDY is in the range of 0.44–1.22 eV [17]. In 2010, Li et al. proposed a new synthetic strategy, which benefited from the development of alkyne complexation, metal-catalyzed cross-coupling and template-assisted synthesis, and successfully prepared GDY on copper substrates by in situ cross-coupling reactions [18]. As a material with a natural band gap, GDY exhibits high electron mobility while being used as an intrinsic semiconductor [19]. GDY has a large nonlinear absorption coefficient, low saturation intensity, broad band Kerr nonlinearity, high nonlinear refractive index and ultrafast relaxation time, all of which indicate that GDY has a long-term prospect in the field of optics.

Here, we demonstrate a femtosecond pulsed laser based on a GDY quantum dot modified tapered fiber. GDY quantum dots are successfully prepared by the Glayser–Hay coupling method. The SA device prepared, based on the nonlinear optical material GDY, has high nonlinear characteristics with 18.06% modulation depth and 103.5 W saturation intensity. The erbium-doped fiber laser achieves an ultrashort pulse output with a spectral width of 26.3 nm and a pulse width of 138.5 fs. Our work highlights the potential of GDY for generating high-performance femtosecond pulses.

## 2. Material Characterization and Device Fabrication

### 2.1. Material

**Figure 1:**
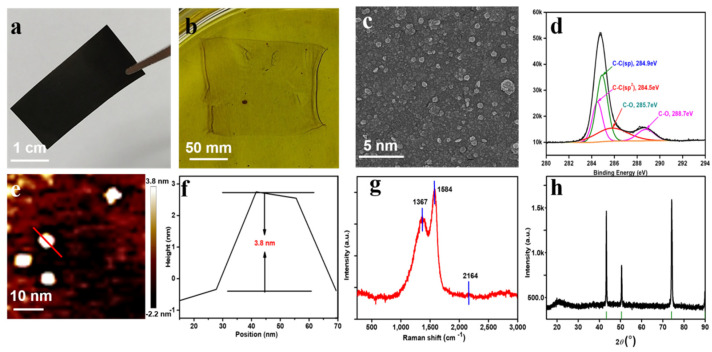
Morphology characterizations of graphdiyne. (**a**) Photograph of the prepared large size GDY on copper foil; (**b**) Photograph of the prepared large size GDY membrane via wet stripping; (**c**) HR-TEM image of graphdiyne, scale bar: 5 nm; (**d**) XPS spectra of graphdiyne film, narrow scan for element C. (**e**) AFM image of the GDY QDs showing the particle thickness. Scale bar: 10 nm. (**f**) The particle size distribution in (**e**), and the average particle size is 3.8 nm. (**g**) Raman spectra of the as-prepared GDY film. (**h**) XRD pattern of graphdiyne QDs.

Graphdiyne is an allotrope of graphite and graphene, which is composed of a lamellar structure of an all-carbon skeleton. The hybridization of carbon elements includes: sp^2^ hybridized benzene ring aromatic system and sp hybridized diacetylene bond. The surface can exist by the functional groups of graphdiyne oxide, and may contain methyl ketone structures in which hydroxyl groups and terminal acetylene bonds are hydrolyzed depending on the synthesis method, storation time and conditions. As shown in Figure 1, the relevant characterization of graphdiyne is shown. Figure 1a shows the large-scale graphdiyne thin films are synthesized on copper foil via the Glaser–Hay coupling reaction reported by Li’s group with hexaethynylbenzene refluxing in pyridine solution at high temperature [18,20]. The plane of graphdiyne supported by copper foil is uniform, smooth and dense. Figure 1b shows the graphdiyne thin film obtained by wet exfoliation of the copper foil-supported graphdiyne in an acidic solution. The thin film is crystal clear, thin and well-proportioned. The transmission electron microscope (TEM) images showed in Figure 1c that the graphdiyne quantum dots were fine-tuned after ultrasonication, centrifugation, alkali cooking, acid washing, and the overall particle size of the graphene quantum dots was well-balanced, and the conduction band electrons, valence band holes and excitons were in three spaces, the direction has been bound in this nanostructure. Figure 1d is the X-ray photoelectron spectroscopy (XPS) spectrum of the graphdiyne nanostructure which shows that the bond energy of the sp hybridized acetylene bond is 284.9 eV, the bond energy of the sp^2^ hybridized aromatic system is 284.5 eV, and the coverage area ratio of the two spectra is about 1:2, in which the presence of C-O bonds indicates that a few parts of the batch of graphdiyne nanomaterials are oxidized. Figure 1e,f show the typical atomic force microscopy (AFM) images of graphdiyne quantum dots with a particle thickness of 3.8 nm. The Raman spectrum (Figure 1g) shows that the C=C double bond stretching vibration of the aromatic system is at 1367 nm^−1^ and 1584 nm^−1^, of which the 2164 nm^−1^ wavenumber corresponds to the stretching vibration of the diacetylenic bond. Figure 1h is the X-ray diffraction (XRD, Cu-Ka radiation) pattern of the nanomaterial, the refraction angle 2θ includes 43°, 51° and 74°, which is consistent with the overall correspondence of the card. In summary, the overall particle size of the batch of graphdiyne nanomaterials is uniform. In ref [21], the linear optical absorption of GDY is measured, and the nonlinear optical properties of GDY are measured by the z-scan method, demonstrating that GDY has strong broadband absorption and strong nonlinear optical properties from the UV to the IR, indicating that it has great potential for applications in photonic devices.

### 2.2. Devices

**Figure 2:**
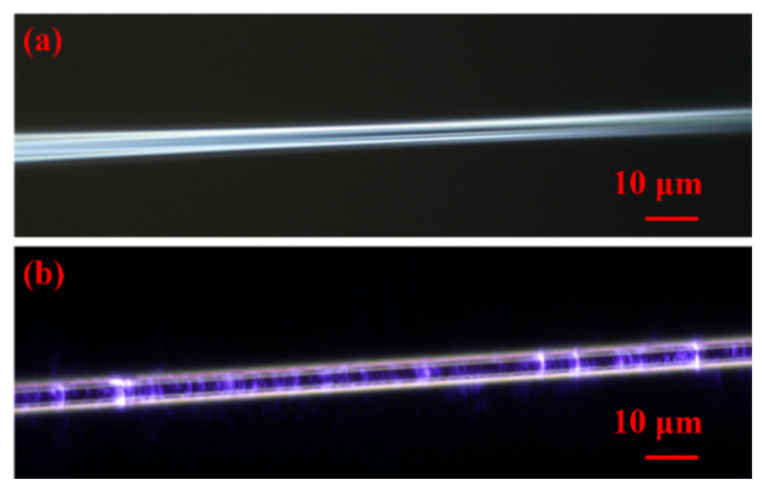
(**a**) tapered fiber; (**b**) GDY-SA: Graphdiyne-modified tapered fiber.

The saturable absorber devices are prepared by the tapered fiber and the saturable absorption characteristics of the tapered fiber-based GDY-SA are measured by the balanced twin-detection method. We used the hydrogen–oxygen flame heating method to prepare tapered fibers based on single-mode fibers YOFC SMF 28. First, the coating layer of the single-mode fiber is peeled off and heated by a hydrogen–oxygen flame, and the fiber is stretched ~10 mm toward both ends at a uniform speed to obtain a tapered fiber with a diameter of ~5 μm (Figure 2a) and a loss of ~0.1 dB @ 1550 nm. The GDY-SA (Figure 2b) is prepared by the optical deposition method based on the coupling mechanism between the near field on the surface of the tapered fiber and the two-dimensional nanomaterial. A 980 nm pump light source is used, and the tapered fiber is connected through an isolator, and then a power meter is connected. With the light source set to 30 mW, the GDY material is dropped onto the tapered area of the fiber through a pipette gun. A microscope is used to observe the attachment of the material to the tapered fiber surface, and the change in power is observed in real time until the material is completely adsorbed and the power is stabilized [22]. Parametric measurements of the prepared devices are performed with deposition losses of ~3 dB @ 1550 nm and ~2.7 dB @ 980 nm.

**Figure 3:**
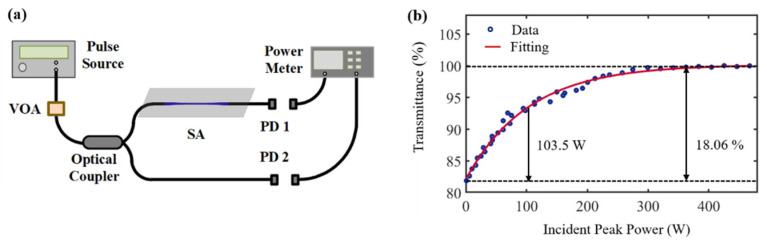
(**a**) balanced twin-detection system; (**b**) Saturation absorption characteristics.

The saturation absorption characteristics of GDY-SA are measured using a balanced twin-detection system (Figure 3a). A pulse source is built as the system light source (a pulse duration of 800 fs, a central wavelength of 1552 nm, and a pulse repetition frequency of 45.9 MHz), and after using an adjustable attenuator, a 1 × 2 50% fiber coupler is used to distribute the outputs; one way is connected to GDY-SA for detection, corresponding to output power P1, and one way is used for power monitoring of the reference signal corresponding to output power P2. Through the continuous adjustment of the attenuator, the last two outputs recorded the power at the output of the device versus the incident light power, the transmittance of the saturable absorber is T = P1/P2. With the increase in peak power intensity, the transmittance of graphdiyne-SA tends to be constant. The nonlinear transmission profile is shown in Figure 3b.

By fitting Eq. $T(I)=1-\Delta T\times \exp(-I/I_{sat})-T_{ns}$, where T(I) is the transmittance, ΔT is the modulation depth (maximum value of the transmission shifted to be 100% but without normalization), I is the incident peak power, $I_{sat}$ is the saturation power, and $T_{ns}$ is the non-saturated loss. The modulation depth ΔT, saturable power $I_{sat}$ and non-saturated loss $T_{ns}$ of GDY-SA are estimated to be 18.06%, 103.5 W and 31.7%. It indicates that graphdiyne-SA has strong saturable absorption performance at 1.5 μm, indicating that the device can be used as an ultrafast optical switch for generating ultrashort pulses at 1.5 μm.

## 3. Experimental Setup and Results

**Figure 4:**
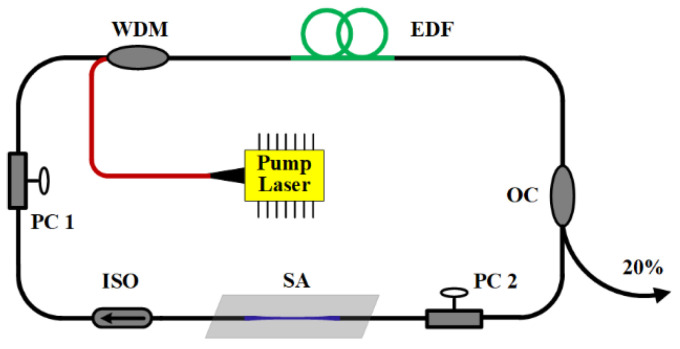
Diagram of an erbium-doped fiber mode-locked pulsed laser based on GDY-SA (WDM: wavelength division multiplexer, EDF: erbium-doped fiber, OC: optical coupler, PC: polarization controller, ISO: isolator).

The saturable absorption characteristics of GDY-SA experimentally demonstrate the saturable absorption characteristics of the device at 1550 nm. The application of GDY-SA to an erbium-doped fiber laser is shown in Figure 4. A 4.3 m erbium-doped fiber (EDF 110) with a group velocity dispersion (GVD) of ~22.7 ps^2^/km @ 1550 nm is used as the gain medium, pumped by a 980 nm laser diode through a 980/1550 nm WDM. The pigtail fiber length at both ends of the WDM is 0.7 m, corresponding to a GVD of −7 ps^2^/km @ 1550 nm. Due to the limitation of the single-mode fiber length in the cavity, extruded PC 1 and PC 2 are selected for adjusting the cavity polarization. The polarization-independent optical ISO ensures unidirectional propagation. The pigtail fiber and connecting fiber for the other devices in the fiber laser cavity is a single-mode fiber with a GVD of −22 ps^2^/km @ 1550 nm. All fibers in the laser cavity consist of positive and negative dispersions, with a total cavity length and net dispersion of ~8.5 m and ~0.016 ps^2^, respectively. The output ratio of the fiber OC is 20:80 and 20% of the output is used for spectral and pulse measurements. The performance of the fiber laser is measured with the aid of an 18 GHz high-speed photodetector using a spectrum analyzer (OSA, YOKOGAWA AQ6370C, Tokyo, Japan), oscilloscope (Agilent MSO7054A, Colorado Springs, CO, USA), RF analyzer (Agilent N9320B, Colorado Springs, CO, USA), and commercial autocorrelator (Femtochrome FR-103, Berkeley, CA, USA).

**Figure 5:**
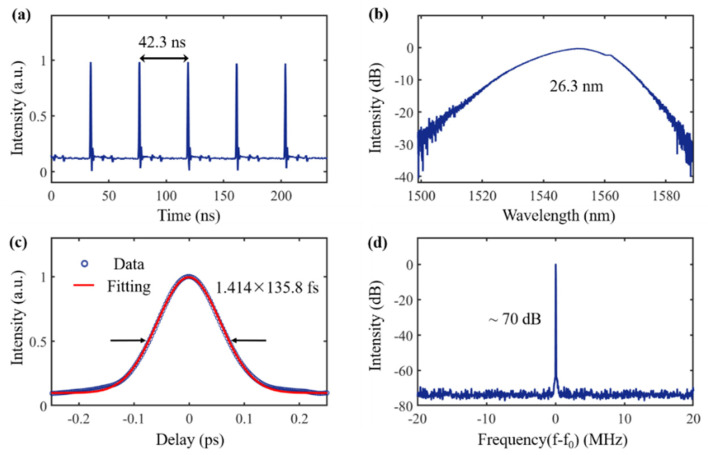
Pulsed output performance of GDY-SA-based erbium-doped mode-locked fiber laser (**a**) typical pulse output sequence with a time interval of 42.3 ns; (**b**) spectrum with a center wavelength of 1551.2 nm and a 3 dB bandwidth of 26.3 nm; (**c**) autocorrelation and Gaussian fit of the output pulse with a pulse width of 135.8 fs; (**d**) RF spectrum with ~70 dB signal-to-noise ratio.

When the pump power is increased to 228 mW, the mode-locking phenomenon can be observed by properly adjusting PC 1 and PC 2. A typical pulse sequence is shown in Figure 5a, with a time interval of 42.3 ns between two pulses, corresponding to a theoretical calculated cavity length of 8.46 m, which is consistent with the actual value. The output power and fundamental repetition frequency of the laser cavity are 1.27 mW and 23.5 MHz, respectively, and the energy of a single pulse is 54 pJ. Figure 5b gives a typical spectrum of the mode-locked ultrashort pulse with a central wavelength of 1551.2 nm and a full width at half maxima (FWHM) of the spectrum of 26.3 nm. The Gaussian fitted autocorrelation curve is shown in Figure 5c, and the pulse duration is ~135.8 fs. The time bandwidth product (TBP) is calculated as ~0.451. The signal-to-noise ratio of the fundamental frequency (f_0_ = 23.5 MHz) spectrum at a resolution bandwidth (RBW of) 1 kHz is ~70 dB with no harmonics as shown in Figure 5d, indicating that the mode-locked fiber laser has good mode-locking performance.

## 4. Discussion

In this work, we prepared the GDY-SA using a combination of graphite and tapered fiber, and in the last decade, a large number of SAs based on 2D nanomaterials have been prepared and applied in photonics to generate ultrashort pulses. The types of SAs can be analyzed in terms of substrates, such as “sandwich” structures, tapered fiber structures, and D-shaped fiber structures. Among them, the device damage threshold of the “sandwich” structure is too low, which limits its application, while the D-type fiber is more expensive and the material integration process is complicated. From the viewpoint of economy, the device damage threshold and complexity of the integration means the tapered fiber is more advantageous.

For the analysis of saturable absorption properties, the measurement of saturable absorption properties of two-dimensional materials often uses Z-scan and balanced twin-detection methods. In this experiment, the balanced twin-detection system is used to more directly verify the saturation absorption properties of the GDY-SA device. According to a large number of reports, the modulation depths are 7.8% [23], 11% [7], 8.8% [24], 10.03% [25], and 11.3% [22] for CNT, Graphene, Bi_2_Te_3_, BP, and MXene materials, respectively. The modulation depth of GDY-SA measured in this work is 18.06%. During the preparation of the GDY-SA device, for the adjustment of the deposition power, the device can withstand a power of 2 W when 980 nm pump light is selected for optical deposition, while for material deposition, only a power of the order of mW is selected to achieve uniform deposition of the material on the surface of the tapered fiber. GDY quantum dots can achieve uniform distribution of material on the tapered fiber surface, and the choice of material is more advantageous compared to conventional SA.

Graphene has more applications in the field of optics, while, graphyne and graphdiyne structures have been very little studied. The 135.8 fs achieved in this manuscript is the shortest pulse in the GDY-SA based erbium-doped fiber laser so far. The performance of GDY-SA is superior to other congener graphene-based absorbers in terms of saturation absorption performance, damage threshold, and pulse width of the fiber.

## 5. Conclusions

In summary, we implemented an ultrashort pulse generated by an erbium-doped fiber mode-locked laser based on GDY-SA. The GDY-based tapered fiber device exhibits strong nonlinear saturable absorption characteristics. Using this GDY-based SA device in an all-fiber erbium-doped mode-locked laser, we demonstrate stable mode-locked operation at 1551.2 nm with a pulse of 135.8 fs and a spectral width of 26.3 nm. The net cavity dispersion of laser is ~0.016 ps^2^. This work highlights the potential of GDY as a member of the graphite family for ultrashort pulse generation in the field of photonics.
